# Pleural Effusion Trajectories and Clinical Outcomes in Cardiac Surgery Patients

**DOI:** 10.31083/RCM37210

**Published:** 2025-06-26

**Authors:** Jun Zhong, Jing-chao Luo, Jin-ling Lin, Ming-hao Luo, Jian Gao, Kai Liu, Guo-wei Tu, Yan Xue

**Affiliations:** ^1^Department of Nursing, Zhongshan Hospital Fudan University, 200032 Shanghai, China; ^2^Cardiac Intensive Care Center, Zhongshan Hospital Fudan University, 200032 Shanghai, China; ^3^Department of Nursing, Zhongshan Hospital Fudan University Xiamen Branch, 361015 Xiamen, Fujian, China; ^4^Department of Biostatistics, Zhongshan Hospital Fudan University, 200032 Shanghai, China; ^5^Department of Critical Care Medicine, Zhongshan Hospital Fudan University, 200032 Shanghai, China; ^6^Shanghai Key Laboratory of Lung Inflammation and Injury, Zhongshan Hospital Fudan University, 20032 Shanghai, China

**Keywords:** pleural effusion, thoracentesis, mortality, cardiac surgical procedures

## Abstract

**Background:**

Pleural effusion (PE) commonly occurs in cardiac surgery patients, often requiring tube drainage. This study aimed to investigate associations between PE drainage trajectories and clinical outcomes in patients undergoing cardiac surgery.

**Methods:**

Patients who underwent cardiac surgery and subsequent tube drainage during hospitalization in the intensive care unit, due to substantial PE, were enrolled. PE drainage volumes were recorded daily. The relationships between PE drainage and poor outcome or mortality risks were examined using logistic regression analysis. Latent class growth analysis (LCGA) was used to classify PE trajectories, and the characteristics of each latent class were compared.

**Results:**

In total, 386 patients were enrolled over 3 years, of whom 113 (29.3%) developed poor outcomes. These patients had significantly higher average PE drainage volumes on days 2–4 (1.7 *vs*. 1.2 mL/kg/day; *p* = 0.002) and days 5–7 (0.9 *vs*. 0 mL/kg/day; *p* < 0.001). Average PE drainage volumes during the first 2–4 and 5–7 days were associated with poor outcomes (odds ratio (OR) = 1.10 (95% confidence interval (CI): 1.02–1.20); *p* = 0.014 and 1.19 (95% CI: 1.08–1.32); *p* < 0.001, respectively). LCGA identified three distinct PE drainage trajectory classes: persistently high (Class 1, n = 39), gradually declining from high to low (Class 2, n = 128), and persistently low (Class 3, n = 219). Among these, Class 1 had the highest mortality and poor outcome risks.

**Conclusions:**

A trend in PE formation demonstrated a strong correlation with mortality and poor outcomes in patients who underwent cardiac surgery. Patients with persistently high PE drainage volumes required close monitoring and attention.

## 1. Introduction

Pleural effusion (PE) is a highly prevalent complication in patients following cardiac surgery, with some medical institutions reporting incidences exceeding 60% [1, 2, 3]. PE is classified into early and late categories based on the time of onset, with early PE occurring within the first postoperative month and accounting for the majority of PE cases after cardiac surgery [4]. PE etiology in this population is multifactorial, including pleural inflammatory reactions induced by cardiopulmonary bypass and surgical trauma, coagulopathy secondary to hemodilution, hypoalbuminemia, and most crucially, elevated capillary hydrostatic pressure resulting from cardiac congestion or suboptimal volume management [2, 4, 5, 6]. Moreover, PE is a significant factor contributing to intensive care unit (ICU) re-admission and poor prognosis outcomes [1, 7].

Minimal pulmonary effusion generally does not warrant further clinical intervention. Conversely, significant pulmonary effusion may induce hypoxia, necessitating thoracentesis to ensure effective drainage [8, 9, 10, 11]. This intervention serves two purposes: it alleviates pulmonary compression and provides an indirect way to assess hydrostatic equilibrium. For the majority of patients, maximum PE volumes are usually drained on the first day. Nevertheless, persistent PE production may require repeated daily drainage.

At present, no studies have analyzed the quantitative relationship between early daily PE drainage trajectories and clinical outcomes. We hypothesize that PE drainage volumes after cardiac surgery are associated with clinical prognosis outcomes, and patients with different PE drainage trajectories have different clinical outcomes. To verify this, we conducted this study.

## 2. Methods

### 2.1 Study Design and Population

This study was part of a cohort study investigating risk factors after cardiac surgery (approved by the ethics committee of Zhongshan Hospital, Fudan University: B2019-075R). From January 2019 to June 2022, in our 40-bed cardiac surgical ICU, patients who underwent thoracentesis (either concurrent bilateral or unilateral) and catheter drainage for large, early PE volumes were included. Inclusion criteria were as follows: (1) undergoing elective cardiac surgery; (2) complete clinical data. Patients were excluded if they met any of the following criteria: (1) aged <18 years; (2) confirmed active infectious diseases; (3) retention of a PE drainage catheter for <7 days due to an unexpected event (catheter dislodgement, premature removal due to discharge or death); (4) severe liver dysfunction or hepatic failure [12]; (5) definitive intrathoracic bleeding caused by surgical procedures; and (6) informed consent was refused by the patient’s legal representative.

Generally, we identified potential patients with large PE volumes using chest radiographs and then assessed volumes using bedside ultrasound. When PE was detected, clinicians first assessed whether it was due to active surgical bleeding which required surgical intervention. Drainage was only performed after excluding the surgical cause of bleeding. Thoracentesis and catheter placement procedures followed in-house protocols [13]. Drainage catheters were either single-lumen central venous (Model CS-24301-E, 16 Ga; Arrow International, Žďár nad Sázavou, Czech Republic; Lot 7175240491; CE 2797) or specialized pigtail drainage catheters (Model DC-0620, 6/8 Fr; Danong Medical, Weifang, China; NMPA Reg. No. 20173140217; Lot C324291204). For patients receiving respiratory support with positive end-expiratory pressure effects, such as high-flow oxygen therapy and non-invasive or invasive mechanical ventilation, the drainage volume was not restricted [14]. Otherwise, drainage volume on the first day was limited to 1000 mL. The daily PE drainage volume was recorded by measuring the amount of bloody fluid in the drainage bag at 6:00 am each day.

### 2.2 Data Collection

We recorded daily PE drainage volumes for the first 7 consecutive days and converted units to mL/kg/day. Additionally, average PE drainage volumes were separately calculated for the first 2–4 and 5–7 days. If the patient had an indwelled drain for <7 days, which was not due to an unexpected event, a volume = 0 was recorded after the drain was removed. Total volume was recorded if patients received bilateral PE drainage (contralateral thoracentesis was performed within 24 h). If not, only the first thoracentesis drainage volume was recorded. Data from electronic medical records included patient demographic information (age, sex, body mass index, chronic disease history), major laboratory tests (N-terminal pro-brain natriuretic peptide (NT-proBNP), total bilirubin (TB), albumin, alanine aminotransferase, aspartate aminotransferase, creatinine, hemoglobin, platelet count, lactate, procalcitonin, white blood cell, etc.), medications (warfarin and aspirin), illness severity scores (European System for Cardiac Operative Risk Evaluation (EuroSCORE) score, Acute Physiology and Chronic Health Evaluation II (APACHE II) score), supportive treatments (renal replacement therapy (RRT), tracheostomy, and non-invasive ventilation), and clinical outcomes (hospital mortality, length of ICU stay, and length of hospital stay).

### 2.3 Outcome Measures

The primary endpoint was a composite endpoint defined as either hospital mortality or postoperative hospitalization exceeding 30 days (poor outcomes). Secondary outcomes included hospital mortality, length of ICU stay, and length of hospital stay.

### 2.4 Statistical Analysis

The normality of continuous variables was assessed using the Kolmogorov-Smirnov test. Data were presented as the mean ± standard deviation for normally distributed variables or the median with interquartile ranges (IQR) for non-normally distributed variables. Categorical variables were expressed as total numbers with percentages. Comparisons were made using Student’s *t*-tests or Wilcoxon rank-sum tests for continuous variables, and chi-square or Fisher’s exact tests for categorical variables, as appropriate. Scatter plots were generated to evaluate parameters potentially associated with PE drainage. Logistic regression analysis was used to assess relationships between PE drainage and poor outcome or mortality risks. Latent class growth analysis (LCGA) was used to classify patients based on their PE volume trajectories, followed by comparisons of clinical characteristics and prognostic differences between patients with distinct PE trajectories. All statistical analyses were performed using SPSS 30.0 software (SPSS Corp., Chicago, IL, USA) and R software (v.4.3.2, R Foundation for Statistical Computing, Vienna, Austria), and a *p*-value < 0.05 was considered statistically significant.

## 3. Results

### 3.1 Patients

During the 3-year study period, 15,928 patients were admitted to our ICU after cardiac surgery, and 386 were included in the study. The cohort comprised of 236 (61.1%) males with a median age of 65 years (IQR = 56–71). Among patients, 40 (10.4%) died during hospitalization, 113 (29.3%) had poor outcomes, 83 (21.5%) underwent tracheostomy, and 80 (20.7%) required RRT support. Median ICU and hospital stays were 8 (IQR = 5–18) and 14 days (IQR = 10–27), respectively. Patients with poor outcomes had higher EuroSCOREs, lower preoperative left ventricular ejection fraction scores, higher preoperative creatinine and NT-proBNP levels before surgery, and also higher APACHE II scores, NT-proBNP levels, TB levels, and lower hemoglobin levels on the day of thoracentesis (Table 1).

**Table 1.** **Clinical characteristics and pleural effusion (PE) in patients with different outcomes**.

		Overall	No poor outcome	Poor outcome	*p* value
		(n = 386)	(n = 273)	(n = 113)	
Preoperative condition					
	Age, years	65 [56–71]	65 [55–71]	65 [57–71]	0.888
	Men, n (%)	236 (61.1)	158 (57.9)	78 (69.0)	0.041
	BMI, kg/m^2^	23 [21–25]	23 [21–25]	23 [21–25]	0.782
	Hypertension, n (%)	189 (49.0)	132 (48.4)	57 (50.4)	0.708
	Diabetes mellitus, n (%)	63 (16.3)	44 (16.1)	19 (16.8)	0.866
	CKD, n (%)	24 (6.2)	17 (6.2)	7 (6.2)	0.990
	EuroSCORE	5 [3–7]	5 [3–7]	6 [4–8]	0.005
	LVEF, %	62 [51–65]	62 [56–65]	60 [45–65]	0.010
	Creatinine, µmol/L	91 [73–121]	88 [72–112]	99 [79–138]	0.002
	NT-proBNP, ng/mL	1154 [378–3123]	918 [332–2616]	1896 [661–4046]	<0.001
Postoperative condition					
	APACHE II score	9 [7–14]	9 [7–13]	11 [8–16]	<0.001
	LVEF, %	58 [49–62]	58 [50–62]	58 [46–63]	0.755
	NT-proBNP, ng/mL	3431 [1471–7975]	2895 [1325–6427]	5292 [2441–12,044]	<0.001
	TB, µmol/L	20 [13–32]	18 [12–30]	22 [15–41]	0.003
	Albumin, g/L	35 [32–38]	35 [32–38]	35 [31–37]	0.190
	ALT, U/L	21 [13–40]	20 [13–35]	25 [12–54]	0.241
	AST, U/L	37 [22–64]	36 [23–58]	38 [20–91]	0.629
	Creatinine, µmol/L	110 [83–171]	108 [85–154]	127 [82–200]	0.147
	Hemoglobin, g/L	86 [76–99]	88 [78–100]	83 [74–98]	0.008
	Platelet, 10^9^/L	107 [73–156]	106 [76–146]	109 [62–178]	0.853
	Lactate, mmol/L	1.6 [1.3–2.6]	1.6 [1.3–2.5]	1.7 [1.3–2.7]	0.378
	Procalcitonin, ng/mL	0.74 [0.31–2.09]	0.66 [0.30, 1.62]	1.16 [0.32, 2.89]	0.009
	WBC, 10^9^/L	10.6 [8.2–13.6]	10.5 [8.3–13.6]	10.9 [8.0–13.8]	0.772
	pH	7.44 [7.41–7.47]	7.44 [7.41–7.46]	7.44 [7.39–7.48]	0.902
	PaCO_2_, mmHg	39 [35–43]	39 [35–43]	37 [34–42]	0.039
	PaO_2_, mmHg	105 [81–147]	103 [80–143]	113 [84–151]	0.173
	Warfarin, n (%)	304 (78.8)	223 (81.7)	81 (71.7)	0.027
	Aspirin, n (%)	104 (26.9)	73 (26.7)	31 (27.4)	0.886
Clinical outcomes					
	Tracheotomy, n (%)	83 (21.5)	19 (7.0)	64 (56.6)	<0.001
	RRT, n (%)	80 (20.7)	31 (11.4)	49 (43.4)	<0.001
	NIV, n (%)	142 (36.8)	97 (35.5)	45 (39.8)	0.426
	Hospital mortality, n (%)	40 (10.4)	0 (0.0)	40 (35.4)	<0.001
	Length of ICU stay, day	8 [5–18]	6 [4–9]	24 [13–39]	<0.001
	Length of hospital stay, day	14 [10–27]	12 [8–17]	40 [32–56]	<0.001
Pleural effusion drainage					
	Day 1, mL/kg/day	11.1 [7.2–16.4]	10.6 [7.1–15.7]	11.8 [7.3–17.3]	0.151
	Day 2, mL/kg/day	2.0 [0.5–4.1]	1.8 [0.5–4.1]	2.2 [0.7–4.2]	0.146
	Day 3, mL/kg/day	0.3 [0.0–1]	0.3 [0–0.9]	0.6 [0.1–1.4]	<0.001
	Day 4, mL/kg/day	0.1 [0.0–0.9]	0.0 [0.0–0.6]	0.5 [0.0–1.5]	<0.001
	Day 5, mL/kg/day	0.0 [0–0.6]	0.0 [0.0–0.3]	0.3 [0.0–1.1]	<0.001
	Day 6, mL/kg/day	0.0 [0.0–0.2]	0.0 [0.0–0.0]	0.1 [0.0–1.0]	<0.001
	Day 7, mL/kg/day	0.0 [0.0–0.1]	0.0 [0.0–0.0]	0.1 [0.0–0.8]	<0.001
	Day 2 to 4 mL/kg/day	1.4 [0.4–3.1]	1.2 [0.4–2.6]	1.7 [1.0–3.8]	0.002
	Day 5 to 7, mL/kg/day	0.0 [0.0–1.3]	0.0 [0.0–0.4]	0.9 [0.0–2.8]	<0.001

Values are median (IQR) or number of patients (n). Poor outcome, defined as either hospital mortality or postoperative hospitalization exceeding 30 days. BMI, body mass index; CKD, chronic kidney disease; LVEF, left ventricular ejection fraction; EuroSCORE, European System for Cardiac Operative Risk Evaluation; APACHE II score, Acute Physiology and Chronic Health Evaluation II score; NT-proBNP, N-terminal pro-brain natriuretic peptide; TB, total bilirubin; ALT, alanine transaminase; AST, aspartate aminotransferase; WBC, white blood cell; RRT, renal replacement therapy; NIV, non-invasive ventilation; ICU, intensive care unit; IQR, interquartile ranges.

### 3.2 PE Drainage Volumes and Associated Factors

**Fig. 1.**
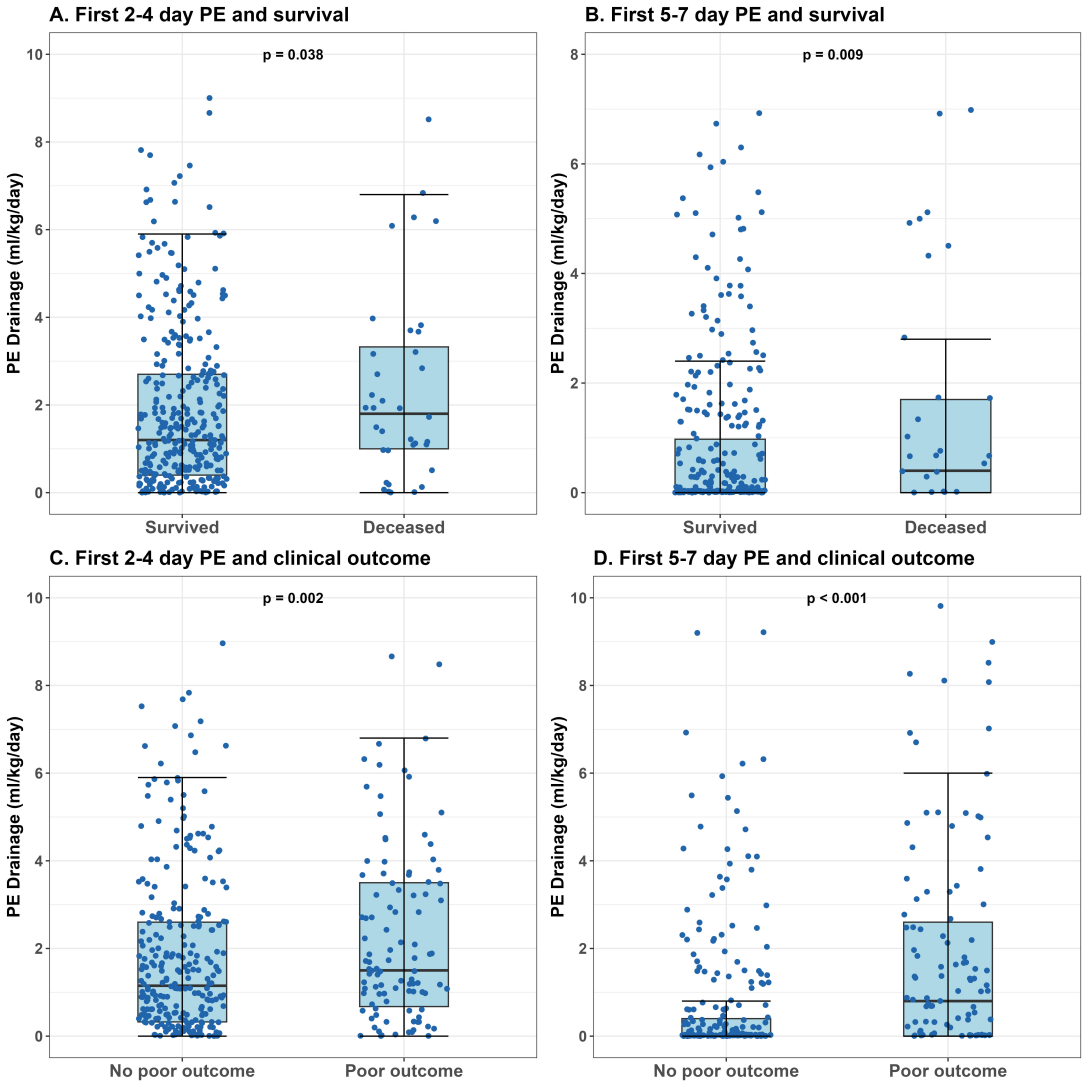
**PE drainage volumes versus patient survival and clinical outcomes**. (A) Significantly higher PE drainage volumes were observed in deceased patients compared to survivors during days 2–4 (*p* = 0.038). (B) Significantly higher PE drainage volumes were observed in deceased patients compared to survivors during days 5–7 (*p* = 0.009). (C) Significantly higher PE drainage volumes were observed in patients with poor outcomes compared to those without during days 2–4 (*p* = 0.002). (D) Significantly higher PE drainage volumes were observed in patients with poor outcomes compared to those without during days 5–7 (*p* < 0.001).

**Fig. 2.**
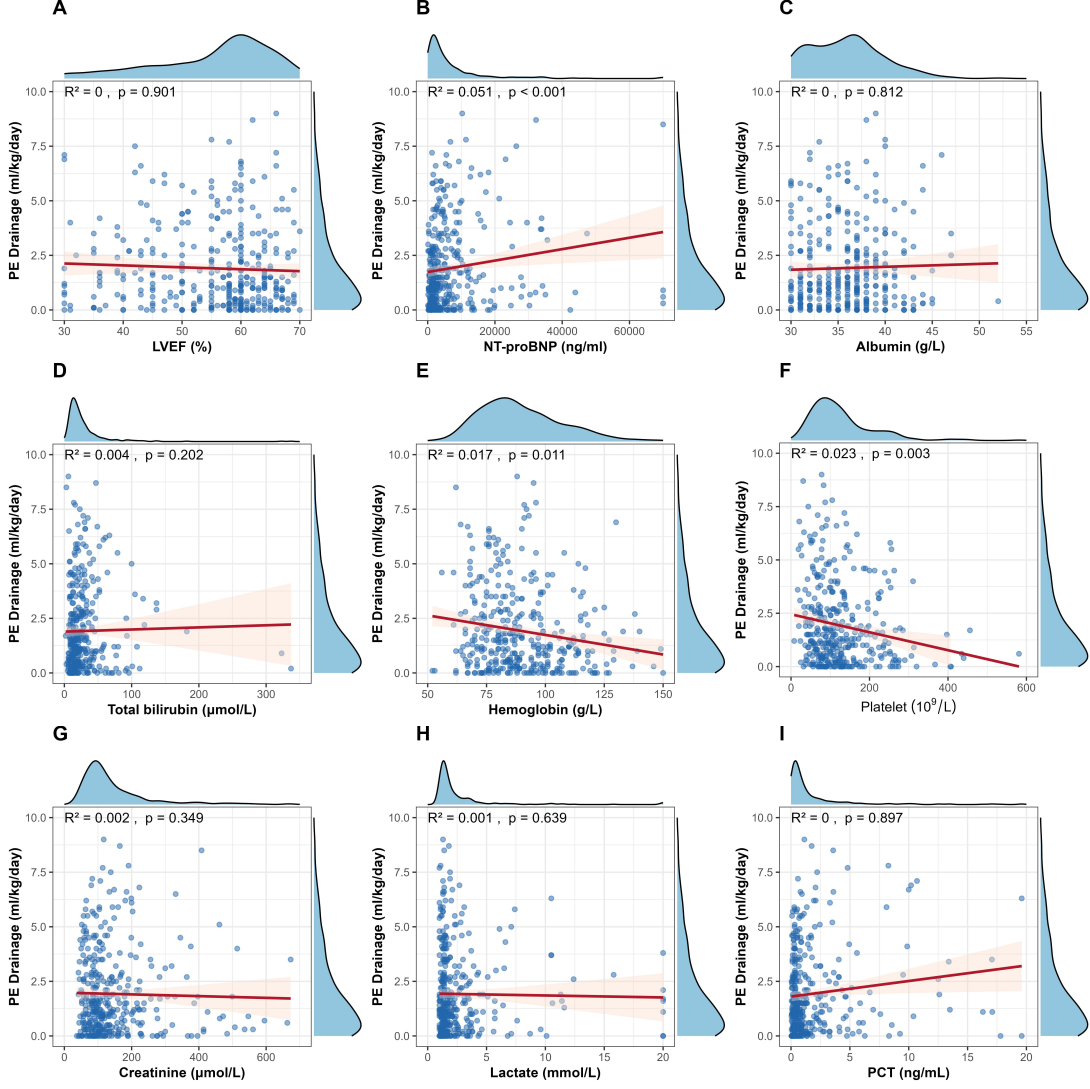
**PE drainage volumes versus laboratory tests on the day of thoracentesis**. (A) PE drainage (mL/kg/day) versus LVEF (%) showed no significant correlation (R^2^ = 0, *p* = 0.901). (B) PE drainage versus NT-proBNP (ng/mL) showed a weak but statistically significant positive correlation (R^2^ = 0.051, *p* < 0.001). (C) PE drainage versus albumin (g/L) showed no significant correlation (R^2^ = 0, *p* = 0.812). (D) PE drainage versus total bilirubin (µmol/L) showed no significant correlation (R^2^ = 0.004, *p* = 0.202). (E) PE drainage versus hemoglobin (g/L) showed a minimal but significant negative correlation (R^2^ = 0.017, *p* = 0.011). (F) PE drainage versus platelet count (10^9^/L) showed a slight but significant negative correlation (R^2^ = 0.023, *p* = 0.003). (G) PE drainage versus creatinine (µmol/L) showed no significant correlation (R^2^ = 0.002, *p* = 0.349). (H) PE drainage versus lactate (mmol/L) showed no significant correlation (R^2^ = 0.001, *p* = 0.639). (I) PE drainage versus procalcitonin (ng/mL) showed no significant correlation (R^2^ = 0, *p* = 0.879). PCT, procalcitonin.

Most PE drainage processes (350 patients, 90.7%) were initiated within 48 h after cardiac surgery, with 279 patients (72.3%) being unilateral (left side: 144 and right side: 135). The median drainage volume on day 1 was 11.1 (7.2–16.4) mL/kg/day, with no significant differences between outcome groups. On day 2, the volume decreased to 2.0 (0.5–4.1) mL/kg/day, but differences remained non-significant. From day 3 onwards, daily drainage volumes were significantly higher in patients with poor outcomes (*p* = 0.001) (Table 1). Patients had higher average drainage volumes in the first 2–4 days (1.7 vs. 1.2 mL/kg/day, *p* = 0.002) and the first 5–7 days (0.9 vs. 0 mL/kg/day, *p* < 0.001) when compared with patients without poor outcomes (Fig. 1). Moreover, a significant difference in PE drainage volume was observed between surviving and deceased patients (*p* < 0.05) (Fig. 1). By analyzing correlations between mean daily PE drainage volumes during post-thoracentesis days 2–4 and laboratory test results obtained on the day of thoracentesis, we found that only NT-proBNP and hemoglobin levels and platelet counts were significantly associated with PE drainage in the first 2–4 days (*p* < 0.05) (Fig. 2).

### 3.3 PE Drainage Volumes and Mortality or Poor Outcome Risks 

The first day’s PE drainage volumes showed no significant associations with hospital mortality (adjusted odds ratio (OR) = 1.03 (95% confidence interval (CI): 0.99–1.07), *p* = 0.170) or poor outcomes (adjusted OR = 1.00 (95% CI: 0.98–1.03), *p* = 0.743) (Table 2). However, the average PE drainage volume in the first 2–4 days showed a stronger, significant association with poor outcomes (adjusted OR = 1.10 (95% CI: 1.02–1.20), *p* = 0.014) (Table 2). This association was further increased in the first 5–7 days (mortality: adjusted OR = 1.13 (95% CI: 1.01–1.26), *p* = 0.036 and poor outcomes: adjusted OR = 1.19 (95% CI: 1.08–1.32), *p* < 0.001) (Table 2). The adjusted factors included EuroSCORE, APACHE II score, NT-proBNP, total bilirubin and warfarin.

**Table 2.** **The relationship between the PE drainage volume and clinical prognosis outcomes**.

Clinical outcomes	Pleural effusion	Crude OR	*p* value	Adjusted OR	*p* value
Death	Day 1	1.04 [1.01–1.08]	0.023	1.03 [0.99–1.07]	0.170
	Day 2 to 4	1.13 [1.04–0.23]	0.006	1.09 [0.96–1.22]	0.066
	Day 5 to 7	1.17 [1.06–0.29]	0.002	1.13 [1.01–1.26]	0.036
Poor outcome	Day 1	1.02 [1.00–1.05]	0.092	1.00 [0.98–1.03]	0.743
	Day 2 to 4	1.15 [1.06–1.24]	<0.001	1.10 [1.02–1.20]	0.014
	Day 5 to 7	1.25 [1.13–1.38]	<0.001	1.19 [1.08–1.32]	<0.001

Data are presented as true value (95% CI). OR, odds ratio.

### 3.4 PE Drainage Trajectories and Their Clinical Meaning

**Fig. 3.**
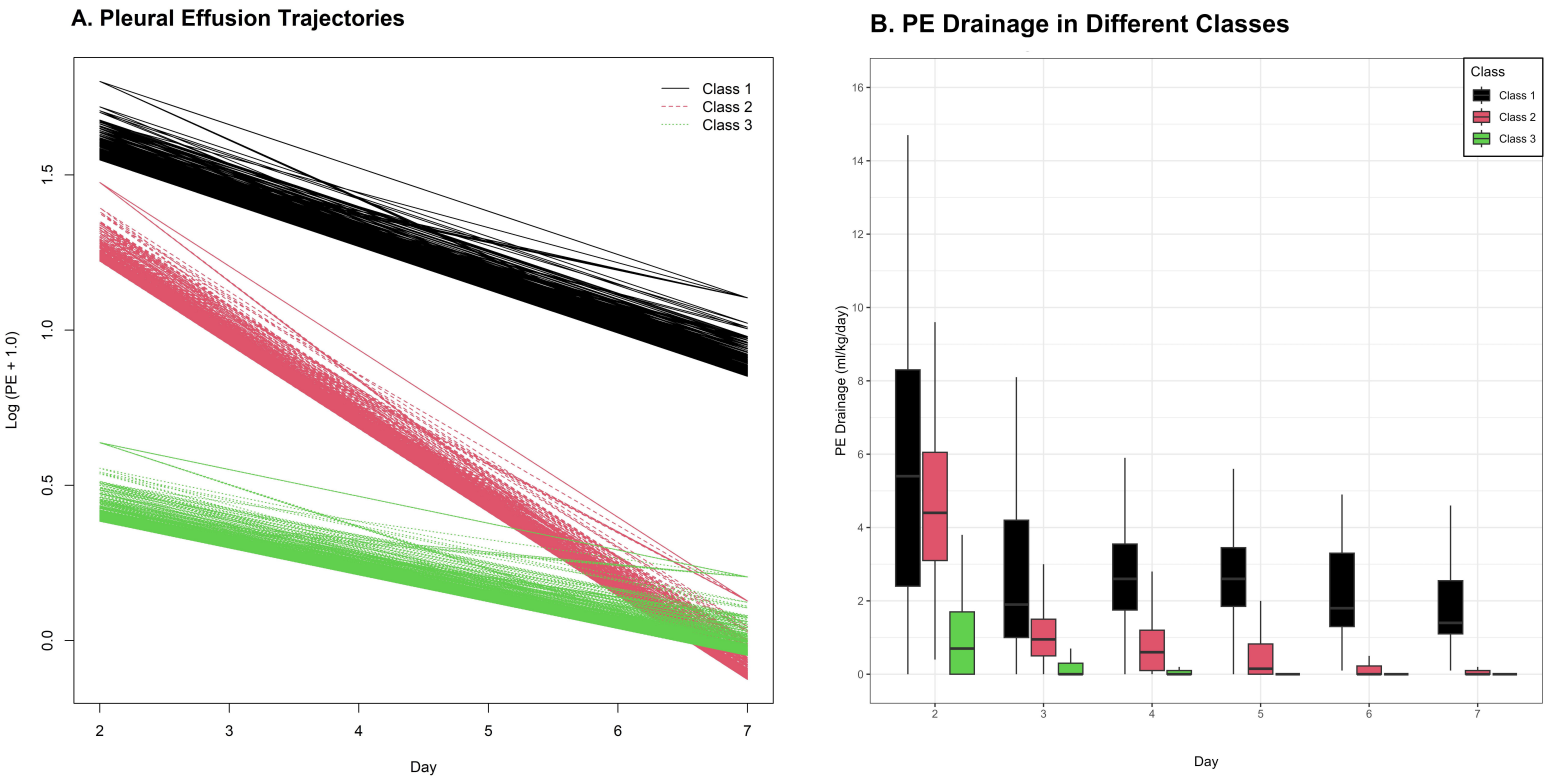
**Three distinct PE drainage trajectory classes**. (A) Three distinct PE drainage trajectory classes identified by latent class growth analysis (LCGA). (B) Box plots of daily PE drainage volume (mL/kg) stratified by trajectory class.

Based on daily PE volumes from days 2 to 7 (after thoracentesis and adequate drainage), PE drainage trajectories were generated. LCGA was used to identify three distinct PE drainage trajectory classes: Class 1 (persistently high levels, n = 39), Class 2 (gradually declining from high to low levels, n = 128), and Class 3 (persistently low levels, n = 219) (Fig. 3). NT-proBNP levels decreased progressively from Class 1 to Class 3 (Table 3). Class 1 patients had the highest hospital mortality (20.5%) and poor outcome rates (53.8%), and the longest ICU (median (IQR) = 15 (9–27 days)) and hospital (IQR = 27 (16–40 days)) stays (Table 3). Although Class 2 patients had slightly higher incidence of poor outcomes than Class 3 patients, the differences were not statistically significant (*p* = 0.306).

**Table 3.** **Different classes of pleural effusion trajectories**.

		Class 1	Class 2	Class 3	*p* value
		(n = 39)	(n = 128)	(n = 219)	
Pleural effusion drainage					
	Day 1, mL/kg/day	20.0 [11.0, 25.6]	14.3 [9.7, 19.6]	8.8 [6.1, 12.9]	<0.001
	Day 2, mL/kg/day	6.3 [2.5, 10.5]	4.5 [3.2, 6.1]	0.7 [0.0, 1.7]	<0.001
	Day 3, mL/kg/day	1.9 [1.0, 4.2]	1.0 [0.5, 1.5]	0.0 [0.0, 0.3]	<0.001
	Day 4, mL/kg/day	2.6 [1.8, 3.6]	0.6 [0.1, 1.2]	0.0 [0.0, 0.1]	<0.001
	Day 5, mL/kg/day	2.6 [1.9, 3.5]	0.2 [0, 0.83]	0.0 [0.0, 0.0]	<0.001
	Day 6, mL/kg/day	1.8 [1.3, 3.3]	0.0 [0.0, 0.2]	0.0 [0.0, 0.0]	<0.001
	Day 7, mL/kg/day	1.4 [1.1, 2.6]	0.0 [0.0, 0.0]	0.0 [0.0, 0.0]	<0.001
	Day 2 to 4 mL/kg/day	5.8 [4.2, 10.8]	2.8 [2.1, 4.5]	0.5 [0.1, 1.1]	<0.001
	Day 5 to 7, mL/kg/day	5.5 [4.3, 8.1]	0.3 [0.0, 1.4]	0.0 [0.0, 0.1]	<0.001
Lab examinations (on the day of thoracentesis)					
	NT-proBNP, ng/mL	5616 [2954, 11,650]	4089 [1647, 8666]	2799 [1326, 6706]	0.002
	TB, µmol/L	28 [14, 44]	21 [14, 29]	18 [12, 32]	0.059
	Albumin, g/L	35 [33, 38]	36 [32, 38]	35 [32, 38]	0.545
	ALT, U/L	18 [12, 28]	20 [12, 40]	22 [14, 42]	0.279
	AST, U/L	39 [21, 72]	36 [22, 68]	36 [23, 60]	0.988
	Creatinine, µmol/L	107 [84, 181]	109 [81, 165]	112 [86, 171]	0.503
	Hemoglobin, g/L	85 [75, 95]	84 [75, 94]	88 [79, 104]	0.011
	Platelet, 10^9^/L	90 [55, 138]	102 [70, 141]	113 [77, 164]	0.046
	Lactate, mmol/L	1.5 [1.4, 3.1]	1.7 [1.3, 2.8]	1.6 [1.3, 2.4]	0.573
	Procalcitonin, ng/mL	0.86 [0.38, 2.30]	0.61 [0.30, 1.77]	0.85 [0.31, 2.42]	0.183
	WBC, 10^9^/L	9.7 [7.6, 13.7]	10.6 [8.6, 13.3]	10.6 [8.3, 14.5]	0.612
Clinical outcomes					
	Hospital mortality, n (%)	8 (20.5)	14 (10.9)	18 (8.2)	0.065
	Length of hospital stay, day	27 [16, 40]	14 [10, 27]	13 [8, 24]	<0.001
	Length of ICU stay, day	15 [9, 27]	8 [5, 16]	7 [4, 15]	<0.001
	Poor outcome, n (%)	21 (53.8)	38 (29.7)	54 (24.7)	0.001

Values are median (IQR) or number of patients (n).

## 4. Discussion 

In this study, we screened more than 15,000 patients, of which 386 had PE with at least one puncture and drainage issue. The most significant conclusion was that increased continuous thoracic drainage was significantly associated with poor patient outcomes after cardiac surgery. To our knowledge, this is the first study to explore the relationship between PE drainage volume (and associated dynamic trajectories) with prognoses in patients undergoing cardiac surgery.

We evaluated associations between PE dynamics and patient outcomes from two perspectives. First, regarding pleural fluid production, higher mean daily PE volumes during postoperative days 2–4 and 5–7 were significantly associated with worse clinical outcomes, indicating that increased fluid production correlated with a poorer prognosis. Second, an analysis of PE production trends revealed that a downward trajectory—whether rapid (resulting in persistently low levels) or gradual—was associated with improved outcomes. In contrast, patients with persistently high PE volumes over 1 week had higher mortality rates and a greater incidence of composite endpoint events. Correlation analyses between PE production rates and cardiac function indices suggested that both the magnitude and duration of postoperative PE potentially reflected underlying cardiac function. In patients with impaired cardiac function, prolonged and elevated PE may have indicated either a lack of therapeutic benefit from the surgical intervention or a sustained procedure-related injury. Notably, these differences in PE dynamics became apparent by postoperative day 3 following thoracentesis and were predictive of subsequent clinical outcomes.

Despite limited studies on PE trajectory, several studies have examined PE causes after cardiac surgery. In Labidi *et al*. [4], patients with very early PEs were hemorrhagic, had higher neutrophil counts, and elevated lactate dehydrogenase levels, and the authors suggested that early effusions were possibly due to pleura trauma, whereas late effusions were more likely due to immune-inflammatory processes related to postcardiac injury syndrome. Pleurotomy can impair the pleura to resorb pleural fluid and may also lead to inflammation contributing to PE formation, consistent with previous findings showing that pleural injury and immunological perturbations were two important PE formation mechanisms after cardiac surgery [15].

Although pleural injury is known to contribute to PE following cardiac surgery, interindividual differences in PE formation rates and duration may also be influenced by other factors. We hypothesize that variations in postoperative cardiac function, particularly left ventricular performance, may have key roles. In heart failure, the primary mechanism underpinning pleural fluid accumulation involves transudation from the pulmonary interstitium into the pleural space. Elevated left atrial and left ventricular end-diastolic pressures are retrogradely transmitted to the alveolar capillaries, promoting fluid leakage and subsequent PE formation. Elevated hydrostatic pressure in these capillaries increases interstitial fluid in the lungs [16]. The fluid then moves from the pulmonary interstitial space across the visceral pleura into the pleural space along a pressure gradient. Pleural fluid accumulates when its formation rate exceeds parietal lymphatic capacity to absorb it.

In this study, we hypothesized that in early PE stages, cardiac insufficiency or excessive circulation load may have triggered PE generation mechanisms. This caused different PE generation rates in different patients from day 3 after pleural puncture and drainage. One observation that could support this hypothesis are NT-proBNP levels. In recent years, circulating natriuretic peptide levels have become useful adjunctive tools in heart failure (HF) diagnostics [17]. Both BNPs and NT-proBNPs are secreted almost exclusively from ventricles in response to pressure and volume overload. Overall, as BNP or NT-proBNP levels increase, the likelihood of HF also increases. In our study, we did not test for BNP in pleural fluid, but collected serum NT-proBNP data. NT-proBNP levels were high in the “persistently high” trajectory group (median (IQR) = 5616 (2954–11,650 ng/mL)) and were associated with mean daily pleural fluid production on days 2–4. Although our analysis revealed a statistically significant correlation between PE drainage volume and serum NT-proBNP levels (R^2^ = 0.051, *p* < 0.001), the low coefficient of determination indicates that this linear relationship accounts for merely 5.1% of the observed variance in drainage volumes. The observed correlation probably reflects the shared underlying pathophysiology of cardiac dysfunction and fluid overload states, consistent with NT-proBNP’s established role as a ventricular stress marker rather than a direct mediator of pleural fluid accumulation.

In our study, another indicator closely related to PE generation was platelet levels. Similar to NT-proBNP, platelets were inversely correlated with average daily PE volumes from 2 to 4 days. Although a statistically significant but weak inverse correlation was observed (R^2^ = 0.023, *p* = 0.003), suggesting that platelet levels may partially reflect disease severity and surgical trauma intensity in PE patients, the minimal explained variance indicates that other pathophysiological factors likely play more predominant roles in determining drainage volumes. In the “persistently high” trajectory group, platelet levels were significantly lower, and levels were highest in the “persistently low” control group. Cardiopulmonary bypass (CPB) used in cardiac surgery may decrease platelet counts in initial phases [18, 19, 20]. Postoperative thrombocytopenia may also occur due to CPB hemodilution and mechanical damage to platelets [21]. In Keles *et al*. [22], pump and cross-clamp times were negatively correlated with platelet counts. Beyond baseline fibrinolysis and inflammatory stimuli associated with blood exposure to cardiopulmonary bypass circuits, high-dose systemic heparin exposure may further increase heparin induced thrombocytopenia perioperative risks [23, 24]. For patients with normal preoperative platelet counts, postoperative decreases may not be necessarily related to cardiac function, but lower platelet counts mean that patients may have higher bleeding risks. In our study, at the time of PE, patients with lower platelet counts had greater pleural fluid production. Therefore, platelet counts may be contributing factors to PE generation and amplify PE effects caused by decreased cardiac function and pleural injury.

## 5. Limitations

This study had several limitations. First, as a single-center, retrospective observational study, the generalizability of our findings may be limited, and the decision to perform thoracentesis and catheter drainage, based on attending physician judgement, may have introduced selection bias. Second, we analyzed PE drainage volumes for only the first 7 days after cardiac surgery, without assessing the long-term impact on patient outcomes or the biochemical/cellular composition of drained PE volumes, which may have highlighted underlying pathophysiology mechanisms. Finally, despite adjusting for potential confounders in logistic regression analysis, residual confounding factors may still have existed due to the observational nature of the study.

## 6. Conclusions

PE formation trends showed a strong correlation with mortality and poor outcomes in patients who underwent cardiac surgery. Patients with persistently high PE drainage volumes represent a population that require close monitoring and attention.

## Availability of Data and Materials

The datasets generated and analyzed during the current study are not publicly available due to restrictions imposed by the hospital’s institutional policies. However, they can be made available from the corresponding author upon reasonable request.
